# Carpal tunnel release with versus without flexor retinaculum reconstruction for carpal tunnel syndrome at short- and long-term follow up—A meta-analysis of randomized controlled trials

**DOI:** 10.1371/journal.pone.0211369

**Published:** 2019-01-28

**Authors:** Sike Lai, Kaibo Zhang, Jian Li, Weili Fu

**Affiliations:** Department of Orthopaedics Surgery, West China Hospital, Sichuan University, Chengdu, People's Republic of China; BG Trauma Center Ludwigshafen, GERMANY

## Abstract

**Background:**

Carpal tunnel syndrome is a common neuropathy disorder for which surgical treatment consists of release and reconstruction of the flexor retinaculum. Reports of postoperative clinical outcomes after carpal tunnel release with or without flexor retinaculum reconstruction in several studies are controversial. This meta-analysis aimed to compare the efficacy and safety of carpal tunnel release with or without flexor retinaculum reconstruction.

**Methods:**

The PubMed, EMBASE, Web of Science, Ovid, Cochrane Library and Clinical Tri Org databases were searched for randomized controlled trials that compared carpal release with and without transverse carpal ligament reconstruction for carpal tunnel syndrome. Outcomes included postoperative Boston Carpal Tunnel Questionnaire Symptom Severity Scale (SSS), Functional Status Scale (FSS), grip strength and complications. The follow-up time was categorized into short-term (0-3mon) and long-term(>3mon).

**Results:**

A total of 7 studies with 613 patients met the inclusion criteria and were analyzed in detail. Statistical analysis showed no significant difference between two groups on postoperative long-term grip strength (MD 5.85, 95% CI -1.05 to 12.76) long-term SSS (MD -0.31, 95% CI -0.75 to 0.13) and occurrence of complications (RR 1.14, 95% CI 0.84 to 1.54), whereas statistically significant difference was found between groups regarding short-term grip strength (MD 1.51, 95% CI 0.86 to 2.17) and long-term FSS (MD -0.34, 95% CI -0.47 to -0.21).

**Conclusion:**

Carpal tunnel release with flexor retinaculum reconstruction for carpal tunnel syndrome may result in improved long-term functional status while there’s no advantage regarding grip strength, symptom severity and safety over individual carpal tunnel release in short- and long-term outcomes.

## Introduction

Carpal tunnel syndrome (CTS) is the most commonly diagnosed compression neuropathy in the upper extremities, which may lead to mild to moderate disability without appropriate treatment[1–3]. The incidence rates reported range from 0.3 to 3.3 per 1000 person per year[4]. Being prevalent in the Medicare patient population, CTS is associated with a large amount of economic burden[5]. The pathophysiology of CTS is complex and results from interactions of many mechanisms. The pathophysiologic mechanism of CTS is likely attributable to abnormally high carpal tunnel pressure and traction neuropathy[6]. Carpal tunnel release (CTR), also described as release of the flexor retinaculum (FR), is the most common surgical technique for CTS. However, complications such as nerve dysfunction, pillar pain and loss of grip strength after CTR have drawn adequate attention. Several modifications of CTR have been introduced to increase the efficacy as well as ensure the safety of treatment, one of which is the flexor retinaculum reconstruction (FRR)[7–11]. However, previous studies comparing effects of CTR with and without FRR show that significant differences do exist, albeit findings are somewhat contradictory[10, 12–15]. Therefore, we set out to perform meta-analysis of the evidence from randomized controlled trials. This study assumed that no difference would be found in grip strength and clinical outcomes between patients undergoing CTR with or without FRR.

## Methods

### Search strategy

This study was designed and conducted according to the guidelines of Preferred Reporting Items for Systematic Reviews and Meta-Analyses (PRISMA) [16]. On June 14, 2018, two independent reviewers searched the PubMed, EMBASE, Web of Science, Ovid, Cochrane Library and Clinical Tri Org databases using the following strategy: ((carpal syndrome OR carpal tunnel syndrome OR CTS) AND (transverse carpal ligament OR transversal carpal ligament OR TCL OR flexor retinaculum OR retinaculum flexorum)). In addition, the references of included articles were screened manually to identify relevant studies

### Inclusion and exclusion criteria

Inclusion criteria were as follows: prospective, randomized controlled trials (RCTs) comparing carpal release with and without flexor retinaculum reconstruction for carpal tunnel syndrome; studies reported in English; studies reported Boston Carpal Tunnel Questionnaire outcomes[17]; and studies measured grip strength using a Jamar hydraulic manual dynamometer. The exclusion criteria were: non-randomized studies; retrospective comparison studies and review studies.

### Data extraction

Two review authors independently extracted data from eligible studies and reached a consensus on all items of the predefined selected form, including first author, year of publication, country or area, number of enrolled participants and number lost to follow-up, mean age and gender of participants, length of follow-up, FRR techniques, the outcome measures and postoperative complications. The follow-up time was categorized into short-term (0-3mon) and long-term(>3mon).

### Methodological quality assessment

The risk of bias for eligible studies were independently assessed by two reviewers following Cochrane recommendations, considering 7 items as follows: random sequence generation, allocation concealment, blinding of participants and personnel, blinding of outcome assessment, incomplete outcome data, selective reporting and other bias[18]. Available protocols of each trial were searched to assess the selective reporting bias. Disagreements were resolved by discussion and consultation to other authors.

### Statistical analysis

Statistical analyses were carried out using Review Manager software (version 5.3; Nordic Cochrane Centre, The Cochrane Collaboration). For continuous outcomes the mean difference (MD) was used as the measure of treatment effect and for dichotomous outcomes the relative risk (RR) was used. Significant heterogeneity referred to where I^2^ value was higher than 50%. A fixed-effects model was initially applied, whereas a random-effects model was used if significant heterogeneity was detected. The 95% confidence intervals were calculated for all cases.

## Results

### Characteristics of included studies

**Fig 1:**
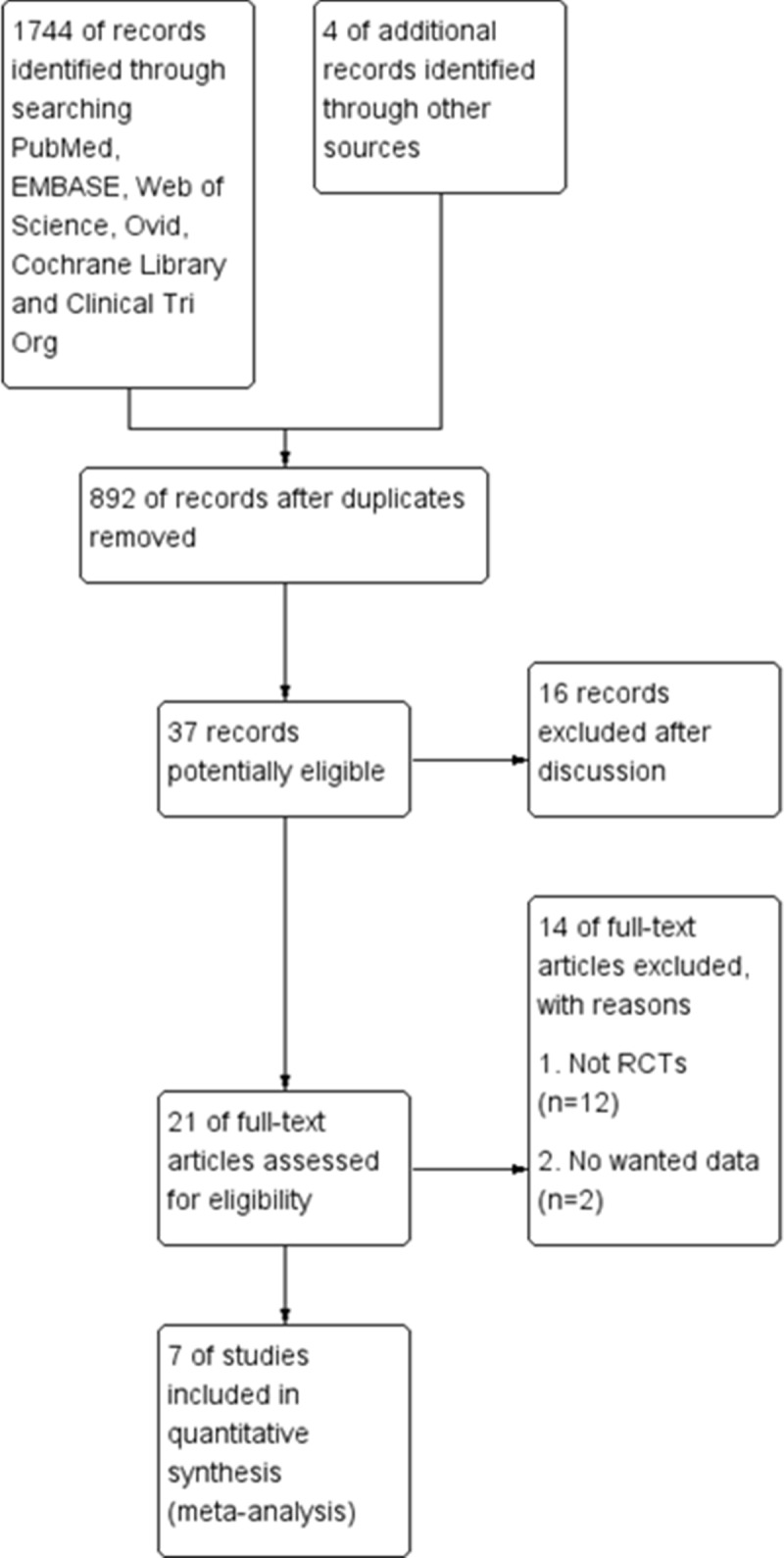
Study flowchart. Five[20, 21, 23–25] of the seven studies used the Simonetta technique to reconstruct the FR, while one study[22] used sub-neural technique and the other one[19] used the Lluch technique. Only one study[22] compared FR reconstruction, open FR release and endoscopic FR release; given that the FR reconstructions were all operated in an open procedure, the endoscopic FR release group was excluded for meta-analysis. Table 1 displays basic characteristics of the pooled RCTs.

As illustrated in Fig 1, a total of 1744 publications were found from the initial search of the online databases and 4 from reference sources. After removal of duplicated abstracts, 892 were left for further evaluation. After titles and abstracts were reviewed, 37 articles seemed to be eligible, 16 of which were excluded after discussion. Full texts of 21 articles were obtained for further screening. Eventually, 7 RCTs [19–25], with 613 patients, were included for further meta-analyses.

**Table 1:** Basic characteristics of pooled studies.

Study	LOE^a^	Country	Reconstruction technique	Sample size		Mean Age		Gender(M/F)		Length of follow-up,mo	Patients Lost to Follow-up,n (%)
				RG^b^	CG^c^	RG	CG	RG	CG		
Dias et al. 2004**[25]**	1	UK	Z-type	26	26	56	56	7/19	7/19	6	0 (0)
Xu et al. 2011**[24]**	1	China	Z-type	34	34	N/A^d^		42/26		12	10 (15)
Faour-Martín et al. 2014**[23]**	1	Spain	Z-type	59	58	49.1	52.2	10/49	6/52	120	5 (4)
Zhang et al. 2015**[22]**	1	China	Sub-neural	68	92	45	47	21/47	33/59	24	0 (0)
Saravi et al. 2016**[20]**	2	Iran	Z-type	21	24	48	51	0/21	4/20	3	7 (13)
Castro-Menéndez et al. 2016**[21]**	2	Spain	Z-type	40	40	48.13		5/35	6/34	12	0 (0)
Gutiérrez-Monclus et al. 2018**[19]**	1	Chile	Ulnar flap	59	58	53.7	54.3	3/56	2/56	6	0 (0)

^a^ LOE, level of evidence

^b^RG, reconstruction group

^c^CG, control group

^d^N/A, not available.

### Assessment of risk of bias

**Fig 2:**
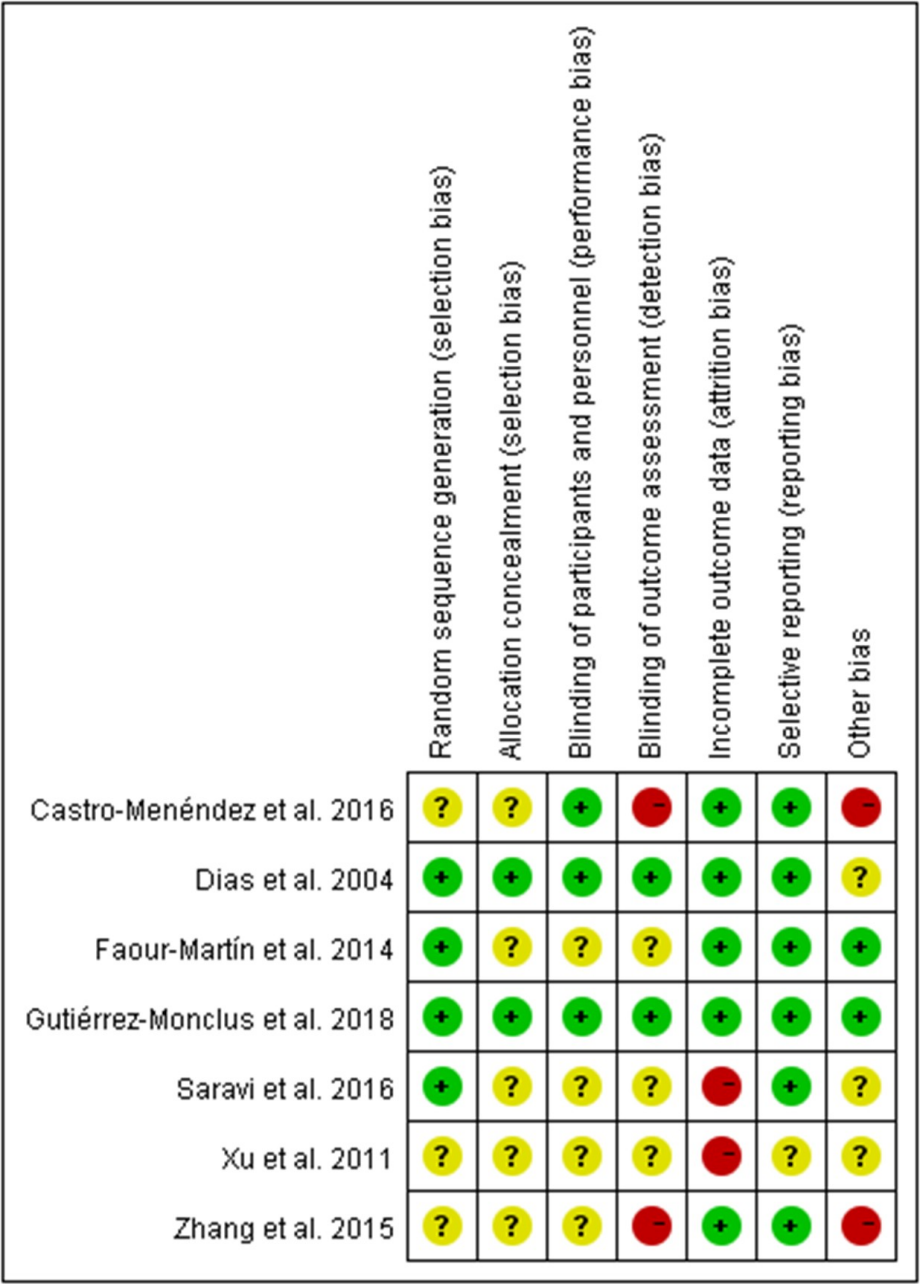
Risk of bias of included studies. +, low risk;–, high risk; ?, unknown risk.

Appropriate random sequence generation was described in four studies[19, 20, 23, 25] (Dias et al. 2004, Faour-Martín et al. 2014, Saravi et al. 2016, Gutiérrez-Monclus et al. 2018). The other three studies[21, 22, 24] (Xu et al. 2011, Zhang et al. 2015, Castro-Menéndez et al. 2016) did not clearly report the methods of randomization. Two studies[19, 25] (Dias et al. 2004 and Gutiérrez-Monclus et al. 2018) conducted adequate allocation concealment while the other five studies[20–24] didn’t describe the method of concealment. Three studies[19, 21, 25] (Dias et al. 2004, Gutiérrez-Monclus et al. 2018, Castro-Menéndez et al. 2016) were rated as having low risk for performance bias for having applied adequate blinding to their participants and personnel, whereas the remained were rated unclear risk at this item for inadequate information. Blinding of outcome measurement was detailed in two studies[19, 25] (Dias et al. 2004 and Gutiérrez-Monclus et al. 2018). Two studies[21, 22] (Zhang et al. 2015, Castro-Menéndez et al. 2016) were graded high risk of performance and detection bias given that the assessors were the same surgeons who had operated on the patients. Two studies[20, 24] were rated high risk of attrition bias due to the high incidence of lost to follow-up (>12%). In the study of Castro-Menéndez et al.[21], the reconstruction procedures were performed by two surgeons while the release procedures were performed by other two surgeons, which may result in other potential bias. Zhang et al.[22] claimed that bias might have arisen owing to the involvement of multiple centers. Fig 2 displays the summary for assessment of risk of bias on pooled studies.

### Grip strength

**Fig 3:**
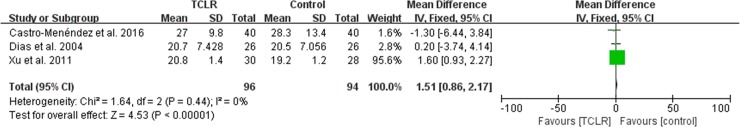
Forest plot diagram showing postoperative grip strength at three months or less compared between the CTR with and without FRR techniques.

Grip strength at 3 months or less postoperatively was reported in 3 studies[21, 24, 25], with 96 patients treated with carpal release with FRR and 94 without FRR as control. As Fig 3 shows, there is a significant difference in favor of FRR (MD 1.51, 95% CI 0.86 to 2.17, I^2^ = 0%, P<0.00001).

**Fig 4:**
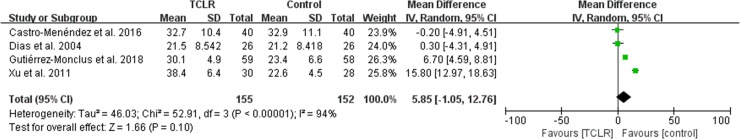
Forest plot diagram showing postoperative grip strength after three months compared between the CTR with and without FRR techniques.

Of the 7 studies, 4 studies[19, 21, 24, 25], including 155 patients treated with FRR and 152 with control, reported long-term outcomes of postoperative grip strength. No significant difference was found between two groups (MD 5.85, 95% CI -1.05 to 12.76, I^2^ = 94%; P = .10) (Fig 4). In consideration of the significant heterogeneity, sensitivity analysis was performed and no statistical difference was shown compared with the original analysis (Table 2).

**Table 2:** Sensitivity analysis.

Study	Parameter	Before Exclusion				After Exclusion				Statistical Significance
		MD^a^	95%CI^b^	Z	P	MD	95%CI	Z	P	
**Xu et al. 2011**	LGS^c^	5.85	-1.05 to 12.76	1.66	0.10	2.62	-2.49 to 7.72	1.00	0.32	No difference
**Gutiérrez-Monclus et al. 2018**	LSS^d^	-0.31	-0.75 to 0.13	1.38	0.17	-0.04	-0.15 to 0.07	0.65	0.52	No difference

^a^MD, standardized mean difference

^b^CI, confidence interval

^c^LGS, long-term grip strength

^d^LSS, long-term symptom score.

## Symptom Severity Scale (SSS)

**Fig 5:**
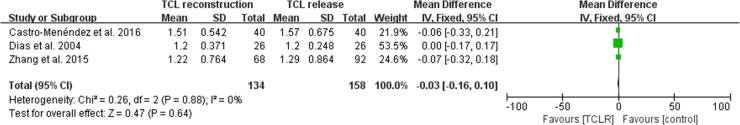
Forest plot diagram showing postoperative SSS at three months or less compared between the CTR with and without FRR techniques.

Of the 7 studies, 3 studies[21, 22, 25], including 134 patients treated with FRR and 158 with control, reported short-term outcomes of SSS. As shown in Fig 5, there’s no statistically significant difference between the groups (MD-0.03, 95% CI -0.16 to 0.10, P = .64, I^2^ = 0%).

**Fig 6:**
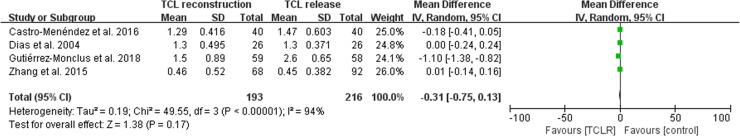
Forest plot diagram showing postoperative SSS after three months compared between the CTR with and without FRR techniques.

Four studies[19, 21, 22, 25], including 193 patients treated with FRR and 216 with control, reported long-term outcomes of SSS. No significant differences between the groups were found after three months (MD -0.31, 95% CI -0.75 to 0.13, I^2^ = 94%; P = .17) (Fig 6). Given the significant heterogeneity, sensitivity analysis was performed and no statistical difference was found compared with the original analysis, indicating that the results are robust. (Table 2)

### Functional Status Scale (FSS)

**Fig 7:**
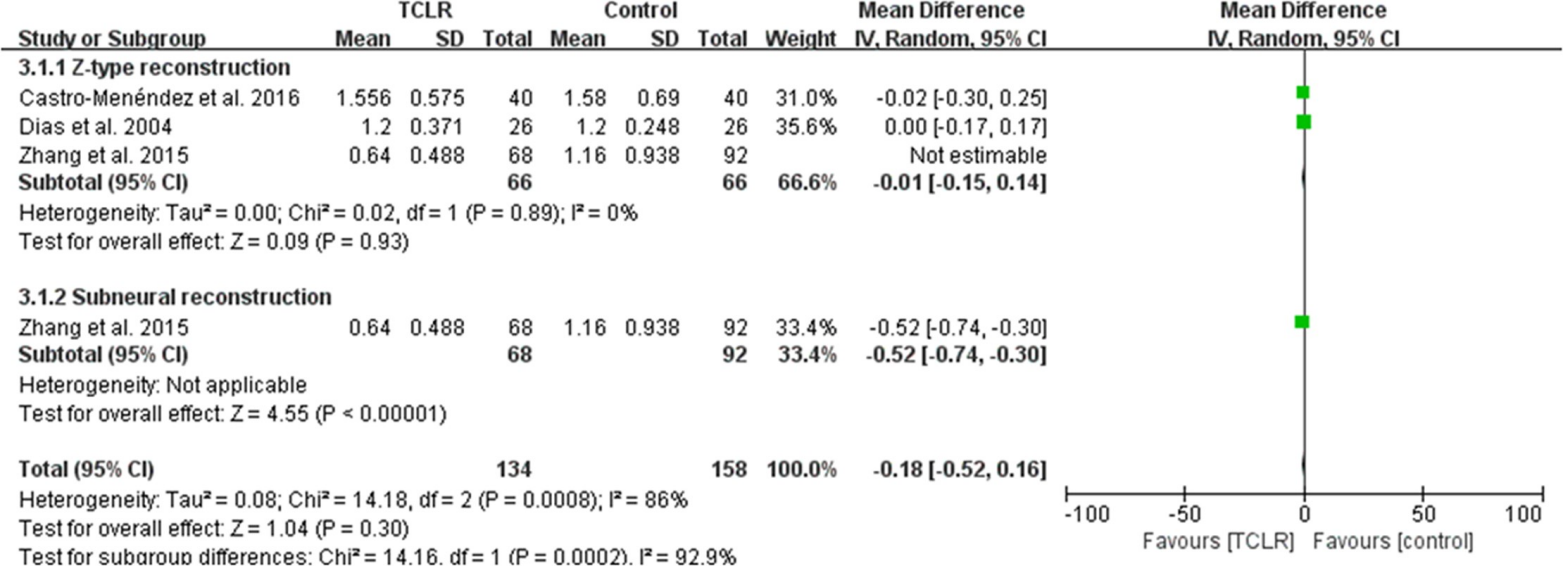
Forest plot diagram showing postoperative FSS at three months or less compared between the CTR with and without FRR techniques, including subgroup analysis by Z-type and sub-neural reconstruction.

Of the 7 studies, 3[21, 22, 25] compared the short-term FSS between groups (MD -0.18, 95% CI -0.52 to 0.16, P = .30, I^2^ = 86%). As can be seen from Fig 7, two studies[21, 25] were assigned to the Z-type reconstruction subgroup, one[22] to the sub-neural reconstruction subgroup. No significant differences between the groups were found in the Z-type subgroup (MD-0.01, 95% CI -0.15 to 0.14, P = .83, I^2^ = 0%). For the sub-neural reconstruction subgroup, the FRR group had statistically significant better outcomes than the control group (MD -0.52, 95% CI, -0.74 to -0.30; P < .00001).

**Fig 8:**
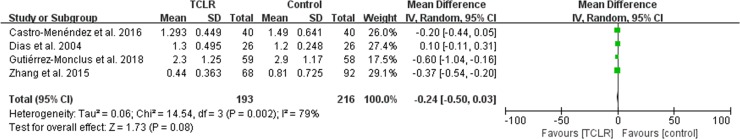
Forest plot diagram showing postoperative FSS after three months compared between the CTR with and without FRR techniques.

Three studies[19, 21, 22, 25], including 193 patients treated with FRR and 216 with control, reported long-term outcomes of FSS. As Fig 8 shows, there is a statistically significant difference in favor of FRR (MD -0.34, 95% CI -0.47 to -0.21, I^2^ = 29%; P < .00001).

### Complications

**Fig 9:**
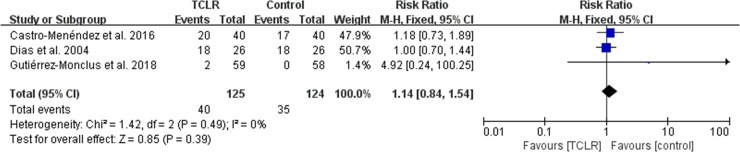
Forest plot diagram showing postoperative complications compared between the CTR with and without FRR techniques.

Of the 7 studies, 3 studies, including 125 patients treated with FRR and 124 with control, reported post-operative complications including pillar pain, paresthesia and scar discomfort. No significant differences between the groups were found (RR 1.14, 95% CI 0.84 to 1.54, I^2^ = 0%; P = .39) (Fig 9).

## Discussion

This meta-analysis did not find a significant difference of short- and long-term outcomes between carpal release with and without FRR regarding symptoms relief and post-operative complications despite a functional benefit of FRR over CTR in short-term grip strength and long-term FSS improvement.

One recent study[26] found that the minimum clinically important difference of grip strength was 6.5kg. Therefore, short-term grip strength improvement of this study may not be clinically significant.

In the pooled studies, the tests were all carried out in 0° position of the wrist according to the standard procedures recommended by the American Society of Hand Therapists[27, 28], in order to maintain the consistency and repeatability. However, the FR acts as a pulley for the flexor tendons and promotes efficiency during finger flexion[29–32]. Therefore, the grip strength measurement should be tested in 30–45° flexion of the wrist in function of the flexor retinaculum. Valid mechanism to maintain a constant wrist flexed position should be developed and applied to grip strength tests[10].

To identify whether the results are robust to assumptions, sensitivity analysis was carried out when heterogeneity was significant (>50%). No statistical difference was detected compared with the original analysis concerning the long-term grip strength and SSS.

Five studies[20, 21, 23–25] included in this meta-analysis used Z-type technique to reconstruct the FR. The original Z-lengthening reconstruction technique was firstly brought out by Simonetta[11], which required a proximal FR flap made by releasing its radial attachment to the carpus and a distal flap developed by releasing the ulnar leaf, as well as preserving its attachment to the hamate hook. One study[21] presented the modified Z-type reconstruction, contrary to the initial technique, it contained a distal flap on the radial side and a proximal flap from the ulnar side. This modification was designed to prevent cutting the insertion of the hamulus in the hamate bone, which may cause pain in the hypothenar eminence[15]. One study[19] conducted the ulnar flap reconstruction by dividing the FR close to the hamate hook and suturing the proximal radial part of the FR to the hamate[33]. The attachment of the FR to the hamulus and pisiform acts as the center of rotation of a joint for the tendons of the flexor digitorum profundus (FDP) and flexor digitorum superficialis(FDS) IV and V[34]. In case of cutting the flexor retinaculum on the ulnar side a dislocation of the flexor tendons of the little finger and ring finger over the hamulus can occur. The other one[22] presented the novel sub-neural procedure by reconstructing the carpal tunnel beneath the median nerve to avoid the risk of recurrent nerve entrapment. To date, several reconstruction procedures have been reported apart from the above all. Netscher et al[14] proposed a FR lengthening technique, suturing the radial based flap to the ulnar distal crease of the divided FR. However, grip strength between groups showed no statistical difference by 12 weeks. Jakab et al[7] divided FR in step-wise fashion and reviewed 73 patients (104 hands), 93% obtained complete relief of symptoms with a minimum follow up of 2 years. Duché et al[35] implanted Canaletto (Eurymed, France) for reconstructing FR. In this prospective cohort study, FRR with this novel implant showed advantages over open CTR concerning of the rapidity of strength recovery and the quality of subjective sensory recovery. There is no literature comparing different FRR technique up to now.

Biomechanical studies have demonstrated a significant increase of carpal arch width between pre- and post CTR[36–41]. Vanhees et al[38] found that sectioning of the FR significantly increased the distal intercarpal distance by 32.9% (p < 0.05), leading to intracarpal mobility increase. Guo et al [42]conducted a finite element study and found the carpal bones located closer to the radius when the FR was released. The axial displacement of the triquetrum decreased more than that of the hamate, resulting in the increased contact stress between these two bones. Schiller et al[41] examined the kinematics changes of the carpus using three-dimensional computed tomography in cadaveric wrists and found significant increase of space between the trapezium and hamate, as well as greater rotation of the hamate around the capitate, along with significant rotation of the pisiform away from the triquetrum. These kinematics changes may explain the post-operative loss of grip strength, pillar pain and palmar tenderness after CTR. Pavlidis et al[43] compared four FR reconstruction techniques regarding the effect on carpal tunnel volume. In this cadaver study, an average increase of carpal tunnel volume ranged from 31% to 44% was found after FR lengthening. It is possible to hypothesize that FR reconstruction is capable of improving the carpal functional status by significantly enlarged the carpal tunnel capacity without interfering its stability. However, no single study exists focusing on the stability and mobility test of carpus in relation of FRR, future studies on the biomechanical topic are therefore recommended.

Another possible cause of the functional status difference is the scar tissue constriction[44–46]. When the FR is cut in longitudinal direction over the median nerve, the median nerve elevates between the two borders of the retinaculum and the scar tissue between the median nerve and the flexor retinaculum leads to nerve constriction. Studies showed traction neuropathy during the repetitive wrist and finger flexion and extension due to the median nerve fixed by scar tissue[47–49].

Being the most common neuropathy disorder in the upper extremities, CTS can lead to a large amount of economic burden. However, very few studies have drawn on any research into the cost-effectiveness of the CTR and FRR procedures. In the current meta-analysis, no single study reported the costs of surgery. Only two studies[20, 25] have recorded cost-related operative time. Significant difference was found in favor of CTR without FRR (MD 6.40, 95% CI 5.31 to 7.49, P < .00001).

It should be noted that there are several limitations of the current study. To begin with, the number of pooled studies is limited due to the language and database restriction. Small sample size (ranging from 45 to 160) of pooled studies potentially has impact on precision of this study. In addition, there are several causes of heterogeneity among pooled studies such as the variability in dominance rate of the affected hand and postoperative rehabilitation protocols. These inherent factors might affect prognosis. Finally, the lack of standardization in the operative technique may have diminished the statistical power of this study.

In conclusion, with support from random controlled trials only, this meta-analysis revealed that carpal release performed with FR reconstruction results in improved long-term functional status. However, FRR has shown no significant advantage of improving grip strength and relieving symptoms in short and long-term follow up. Further researches could be conducted regarding the biomechanical test and cost-effectiveness investigation.

## Supporting information

S1 PRISMA checklist (DOC)
